# Physicochemical Assessment of Plant-Derived Residues as Potential Components of Substrates for *Pleurotus ostreatus* Cultivation

**DOI:** 10.3390/ma19194127

**Published:** 2026-09-27

**Authors:** Kinga Koziak, Jolanta Piekut, Małgorzata Krasowska, Izabela Zgłobicka, Ewa Zapora

**Affiliations:** 1 Department of Agri-Food Engineering and Environmental Management, Faculty of Civil Engineering and Environmental Sciences, Bialystok University of Technology, Wiejska St. 45E, 15-351 Białystok, Poland; j.piekut@pb.edu.pl (J.P.); m.krasowska@pb.edu.pl (M.K.); 2 Department of Materials and Production Engineering, Faculty of Mechanical Engineering, Bialystok University of Technology, Wiejska St. 45C, 15-351 Białystok, Poland; i.zglobicka@pb.edu.pl; 3 Department of Silviculture and Forest Utilization, Faculty of Civil Engineering and Environmental Sciences, Bialystok University of Technology, Wiejska St. 45E, 15-351 Białystok, Poland; e.zapora@pb.edu.pl

**Keywords:** plant-derived residues, mushroom cultivation, oyster mushroom, sustainable development, circular economy

## Abstract

Plant-derived residues may provide locally available components for mushroom cultivation substrates, but their variable composition requires preliminary physicochemical characterization. The aim of this study was to evaluate the physicochemical parameters of selected plant-derived residues in terms of their suitability as substrate components for the cultivation of *Pleurotus ostreatus*. The primary raw materials used were: oak sawdust, birch sawdust, wheat straw, triticale straw, rapeseed straw, and hemp shives. In addition, spent coffee grounds and apple pomace were applied as supplements enriching the substrate. The tested materials were subjected to physicochemical analyses including the determination of pH value, nitrogen, phosphorus, potassium, magnesium, calcium, sodium, organic carbon, and heavy metal content. The lignocellulosic materials showed high C:N ratios (80–257), indicating the potential need for nitrogen supplementation. Spent coffee grounds had the highest nitrogen content (21.61 g·kg^−1^ DM) and the lowest C:N ratio (25.96), while apple pomace showed the lowest pH (3.85). Wheat and triticale straw supplemented with spent coffee grounds were identified as promising candidates for further cultivation trials, supporting the valorization of plant-derived residues in line with circular economy principles. As the study was limited to preliminary physicochemical screening, lignocellulosic-fraction analysis, physical characterization, and cultivation trials are required before the technological suitability of the proposed mixtures can be confirmed.

## 1. Introduction

Progressive environmental degradation and growing challenges associated with the management of residues generated by the agri-food industry have increased interest in solutions aligned with the concept of the circular economy (CE). This model assumes the reintegration of by-products into the production cycle in order to reduce environmental pressure and improve resource-use efficiency [1,2,3]. Properly characterized and processed plant-derived residues may constitute valuable secondary raw materials. Their safe and technologically justified utilization is therefore an important component of sustainable production systems [4,5].

According to data from FAO and Eurostat, a substantial proportion of plant biomass remains unused or is managed through low-value applications, generating additional environmental and economic costs. The effective incorporation of these streams into substrates for mushroom cultivation may significantly enhance their material valorization [6,7].

Within the circular-economy framework, mushroom cultivation represents a biological valorization pathway through which lignocellulosic residues can be converted into fungal biomass and potentially reusable spent substrate. One of the promising directions for the utilization of plant-derived residues is their application as substrates in the cultivation of edible mushrooms. Species of the genus *Pleurotus* possess the ability to efficiently degrade lignocellulosic materials due to the activity of ligninolytic enzymes such as laccases and peroxidases [8]. *Pleurotus ostreatus (Jacq.) P. Kumm.* is a saprotrophic basidiomycete widely distributed in temperate climatic zones, naturally colonizing dead hardwood. It is among the most extensively cultivated edible mushrooms worldwide and accounts for approximately 15–25% of global mushroom production. This species is highly valued because of its short production cycle, relatively low technological requirements, and ability to grow on a wide range of lignocellulosic substrates [9,10].

In addition to its economic importance, *Pleurotus ostreatus* is characterized by high nutritional value. Its fruiting bodies contain protein with a favorable amino acid profile, dietary fiber, and bioactive compounds, including polysaccharides with antioxidant and immunomodulatory properties [8,9]. The growing popularity of plant-based diets and the search for alternative protein sources further increase the significance of edible mushroom production within sustainable food systems [10].

The cultivation technology of oyster mushroom is based primarily on the use of lignocellulosic substrates derived from agricultural and woody residues, such as cereal straw, hardwood sawdust, rice husks, corn cobs, and sugarcane bagasse [8,9,11]. The suitability of these materials depends both on their chemical composition and physical properties. The contents of lignin, cellulose, and hemicellulose, the carbon-to-nitrogen ratio (C:N), pH, moisture content, and the physical structure of the substrate significantly affect the rate of mycelial colonization and biological efficiency [12].

The C:N ratio is considered one of the key parameters determining mycelial growth and yield formation. Carbon is the primary source of energy, whereas nitrogen is essential for the synthesis of proteins and enzymes. Excess nitrogen may increase the risk of contamination, while nitrogen deficiency limits mycelial development [13]. The literature reports favorable C:N values of approximately 20:1–30:1 [8,12] as well as higher values of approximately 45:1–60:1 [14,15]. Higher biological efficiency has also been reported within a C:N range of 38:1–58:1, with the best results in the narrower range of 38:1–48:1 [16]. Accordingly, 25:1–60:1 should be interpreted as a broad screening interval rather than a single optimum: values of 25:1–45:1 are preferred during preselection, whereas values above 45:1 and approaching 60:1 are boundary values requiring biological validation. The C:N ratio alone cannot determine substrate suitability because substrate performance is also affected by nitrogen source and availability, lignocellulosic composition, moisture content, particle size, porosity, and substrate processing [12,17].

Substrate pH is also of considerable importance. The best mycelial growth is generally observed under slightly acidic conditions, within a pH range of approximately 5.0–6.5, although in commercial practice values close to neutral are often applied in order to stabilize conditions and limit the development of competing microorganisms [12,18].

The physical properties of the substrate are equally important. Adequate porosity and structural stability ensure proper water retention and oxygen availability, enabling correct metabolic processes and uniform colonization of the substrate by the mycelium. Excessive particle size reduction may lead to compaction and restricted gas exchange, whereas overly coarse fractions may hinder colonization [12].

In addition to carbon and nitrogen, mineral nutrients such as phosphorus, potassium, calcium, and magnesium are also important, as they participate in energy transformations, water balance regulation, and stabilization of cellular structures. Their availability affects enzymatic activity and overall biological efficiency [9]. In the case of non-standard substrates derived from agri-food residues, it is therefore particularly important to consider both lignocellulosic composition and the balanced supply of macro- and micronutrients [17].

Despite numerous studies concerning the use of various plant residues in oyster mushroom cultivation, the suitability of specific materials depends to a large extent on their physicochemical properties, preparation method, and proportion in the substrate mixture. The local availability of raw materials further justifies the need for their individual characterization prior to implementation in production practice [11,17,19].

Fully colonized lignocellulosic substrates are also being investigated as mycocomposites for potential applications in construction, packaging, and insulation materials [20,21].

Previous studies have evaluated individual agro-industrial residues as substrates for mushroom cultivation; however, systematic comparisons of locally available residues with a commercial reference substrate using a unified physicochemical screening and mixture-design framework remain limited. The novelty of this study lies in the integrated comparison of locally sourced woody, agricultural, and food-processing residues with a commercial *Pleurotus ostreatus* substrate using a unified analytical protocol. Elemental characterization was combined with dry-mass mixture calculations and Solver-assisted screening to identify candidate formulations for subsequent biological validation.

The aim of the present study was to characterize the physicochemical properties of selected, locally available plant-derived residues (woody, agricultural, and food-processing residues) and to conduct a preliminary evaluation of their potential as components of substrates for *Pleurotus ostreatus* cultivation. The obtained results may provide a basis for further considerations regarding their potential application in substrate production technology, in accordance with the principles of the circular economy.

## 2. Materials and Methods

### 2.1. Materials

The research material consisted of plant-derived residues obtained in the third quarter of 2025 from local farms and agri-food- and wood-processing enterprises located in Siemiatycze County, Podlaskie Voivodeship, Poland. Spent coffee grounds were collected from a café in Białystok, Poland. A commercially available substrate of undisclosed composition was used as a practical reference material for *Pleurotus ostreatus* cultivation. Because the residues were obtained from multiple entities and locations, a single source city could not be assigned to all materials. The supplier/manufacturer of the commercial reference substrate is not disclosed. Several batches were obtained for selected materials, particularly straw and spent coffee grounds. Depending on the material, the total collected amount ranged from several to more than ten kilograms. Primary samples from the available batches of a given material were combined and thoroughly homogenized to obtain one composite sample for each material. Subsamples were then collected from each composite sample for three analytical replicates (n = 3). The results therefore characterize the investigated composite samples and do not constitute a comprehensive assessment of the regional variability of each residue type.

The investigated materials were divided into the following groups (Figure 1a–i):

a) Commercially available substrate used as a practical reference material (a).

b) Selected plant-derived residues, including:

•Woody residues, including: oak sawdust (b) and birch sawdust (c);

•Agricultural residues, including: wheat straw (d), triticale straw (e), rapeseed straw (f), and hemp shives (g);

•Food industry residues, including: apple pomace (h) and spent coffee grounds (i).

### 2.2. Methods

All tested raw materials were subjected to preliminary preparation prior to laboratory analyses, including drying to constant mass, grinding, and homogenization in order to obtain uniform samples and ensure comparability of results.

All materials were dried at 105 °C until constant mass was achieved before pH determination and all elemental analyses. This temperature was used to determine dry matter, standardize sample condition, and express analytical results on a dry-mass basis. The dried material was then ground to a homogeneous fraction of approximately <0.5 mm. The prepared samples were stored in sealed containers at room temperature until analysis.

The total organic carbon (TOC) content was determined using a Shimadzu TOC-L analyzer equipped with an SSM-5000A solid sample combustion unit (Shimadzu Corporation, Kyoto, Japan). The prepared solid samples were analyzed by high-temperature combustion, and the carbon content was quantified by infrared detection of the generated CO_2_.

The total nitrogen content was determined by the Kjeldahl method in accordance with PN-G-04523:1992 [22]. Approximately 0.5 g of the ground sample was digested in concentrated sulfuric acid, and the resulting solution was subsequently subjected to distillation and titration using a Vapodest 50S apparatus (Gerhardt, Königswinter, Germany). Results were expressed on a dry matter basis.

The C:N ratio was calculated on a dry-mass basis as the quotient of the mean total organic carbon content and the mean total nitrogen content expressed in equivalent mass units. For each mixture, total carbon and total nitrogen contents were calculated as the dry-mass-weighted sums of the corresponding measured contents in its components, and the mixture C:N ratio was obtained by dividing the calculated total carbon content by the calculated total nitrogen content. Carbon and nitrogen were each determined in three analytical replicates, and their variability is reported separately as standard deviations. Because C:N was derived from the mean values rather than measured independently, it was used descriptively and was not assigned a separate standard deviation or subjected to analysis of variance.

The contents of sodium (Na), potassium (K), magnesium (Mg), and calcium (Ca) were determined using a Thermo Scientific iCE 3300 flame atomic absorption spectrometer (Thermo Fisher Scientific, Waltham, MA, USA). Calibration curves were prepared using standard solutions of Na, K, Mg, and Ca at concentrations ranging from 0.5 to 100 mg·L^−1^. Subsequently, the concentrations of macroelements in the tested mineralizates were determined.

The contents of phosphorus (P), iron (Fe), zinc (Zn), copper (Cu), chromium (Cr), nickel (Ni), cadmium (Cd), mercury (Hg), lead (Pb), and arsenic (As) were determined using inductively coupled plasma mass spectrometry (ICP-MS) with an 8800 Triple Quadrupole ICP-MS spectrometer (Agilent Technologies, Santa Clara, CA, USA).

The pH was determined potentiometrically using samples previously dried at 105 °C and ground as described above. An aqueous suspension was prepared at a 1:5 sample-to-water volume ratio, mixed for 60 ± 10 min, and left to equilibrate for approximately 3 h. The pH was measured using a pH meter equipped with a glass electrode (Mettler-Toledo GmbH, Greifensee, Switzerland) after the reading had stabilized. The suspension preparation and measurement procedure were based on PN-EN ISO 10390:2022-09 [23].

All analyses were performed in three analytical replicates (n = 3), and the results are expressed as mean values with standard deviation. Statistical analysis was carried out using Statistica 13.3 software (TIBCO Software Inc., Palo Alto, CA, USA). One-way analysis of variance (ANOVA) was performed, followed by Tukey’s honestly significant difference (HSD) post hoc test. Differences were considered statistically significant at *p* ≤ 0.05.

Based on the experimental data, radar charts were prepared using Microsoft Excel 16.0 (Microsoft Corporation, Redmond, WA, USA). Min–max normalization was selected to transform variables expressed in different units to a common 0–1 scale while preserving their relative ordering within the analyzed dataset. The normalized values were used solely for descriptive visualization and were not used for statistical inference, mixture calculations, or Solver analysis.

To evaluate the possibility of obtaining a substrate with physicochemical parameters suitable for the cultivation of *Pleurotus ostreatus*, component proportions were analyzed using the Solver add-in in Microsoft Excel 16.0. The analysis was treated as a preliminary screening approach, and two-component mixtures consisting of one base material and one additive were considered on a dry-mass basis. A C:N range of 25–60 was adopted as a broad screening interval rather than a definitive cultivation optimum [8,12,13,14,15,16,18]. Values of 25–45 were treated as preferred during preselection, whereas values above 45 and approaching 60 were treated as an extended screening range requiring biological validation. Because C:N is only one of several determinants of substrate performance, the screening result does not account for nitrogen form, lignocellulosic composition, physical structure, moisture, or processing effects. A maximum additive content of 20% (*w*/*w*, dry-mass basis) was adopted as a practical preselection criterion, not as a biologically confirmed optimum. This limit was intended to reduce the risk of excessive changes in substrate structure and acidity and excessive inputs of readily available compounds, phenolics, caffeine, and lipids from spent coffee grounds. The Solver analysis identified feasible component proportions satisfying the adopted C:N criterion within this limit. Mixtures containing 25% and 30% spent coffee grounds were included solely as comparative variants exceeding the limit and were not considered formulations meeting all screening criteria. The mixtures were prepared on a dry-mass basis and analyzed before pasteurization, sterilization, or incubation. Water was added only to prepare the analytical pH suspension; the mixtures were not hydrated to cultivation moisture or incubated before measurement.

## 3. Results and Discussion

Based on the total carbon (C) content, total nitrogen (N) content, and C:N ratio (Table 1, Figure 2), the materials were classified into two functional groups:

•Base components—lignocellulosic materials with high carbon content and a structural function,

•Supplements—components with elevated nitrogen content and greater supplementation potential.

The base components included sawdust (oak and birch), straws (wheat, triticale, and rapeseed), and hemp shives. These materials were characterized by high carbon content ranging from 469.55 to 522.80 g·kg^−1^ DM and low nitrogen content (2.035–5.850 g·kg^−1^ DM), resulting in high C:N ratios ranging from 80.27 (triticale straw) to 256.90 (birch sawdust). These values were descriptively higher than the C:N ratio of the commercial reference substrate (49.05), indicating the potential need for nitrogen supplementation if these materials are used as dominant substrate components. As reported by Bellettini et al. (2019) [17], maintaining an appropriate carbon-to-nitrogen ratio is one of the key factors affecting fungal enzymatic activity and biological efficiency. The authors suggested supplementing the substrate with cereal bran to increase nitrogen content [17]. Other researchers have likewise emphasized the necessity of enriching lignocellulosic substrates with nitrogen [24,25]. Kumla et al. (2020) indicated cereal husks and manure as potential supplements and Miśkiewicz et al. (2025) proposed brewer’s spent grain at levels up to 30%, whereas Chai et al. (2021) investigated the use of spent coffee grounds at approximately 10–30% [25,26,27]. Mkhize et al. (2022) highlighted that nitrogen supplementation is essential to achieve a C:N range favorable for species of the genus *Pleurotus* [28].

The conducted analyses showed that spent coffee grounds exhibited the highest nitrogen content (21.610 g·kg^−1^ DM) and the lowest C:N ratio (25.96), considerably lower than that of the commercial substrate. This indicates their potential value as a nitrogen-rich supplement. Apple pomace contained 6.525 g·kg^−1^ DM of nitrogen, and its C:N ratio was 70.36, higher than that of the commercial substrate but lower than in most lignocellulosic materials. Thus, apple pomace should be considered primarily as an acidic fruit-processing residue contributing phosphorus and some carbon and nitrogen, rather than as a typical nitrogen supplement.

The pH of the analyzed materials ranged from 3.85 to 6.14. The lowest pH was recorded for apple pomace (3.85), which was below the adopted target pH range for *Pleurotus ostreatus* substrate formulation (5.0–6.5). The materials closest to the optimum were rapeseed straw (6.14) and the commercial substrate (5.71). This indicates that a higher proportion of apple pomace in the mixture would require pH adjustment. Sawdust materials were characterized by pH values ranging from 4.45 to 4.63, also below the optimum for this species, suggesting the need for pH regulation in mixtures containing high proportions of sawdust.

The literature emphasizes the necessity of monitoring and, where necessary, correcting substrate pH, particularly when acidic supplements are used [10,29]. Published data indicate that the addition of spent coffee grounds may increase substrate acidity and the content of phenolic compounds [30]. At the same time, owing to their high nitrogen content and low C:N ratio, they significantly affect the elemental balance of the mixture. This means that their proportion in the substrate should be precisely controlled, as they modify both pH and the C:N balance.

The normalized radar chart (Figure 2) confirmed the contrasting physicochemical profiles of the analyzed materials. Spent coffee grounds were distinguished by high nitrogen content and low C:N ratio, whereas sawdust materials showed the highest C:N values. Straw materials and hemp shives occupied an intermediate position, supporting their potential role as structural components requiring supplementation. The normalized values show relative physicochemical profiles only and should not be interpreted as substrate-suitability scores.

The analysis of macronutrient content (Table 2, Figure 3) revealed clear descriptive differences among the material groups. Substrates based exclusively on lignocellulosic raw materials were characterized by relatively low mineral nutrient content, which may limit later cultivation performance and justifies considering supplementation in further trials. Enrichment of the substrate with macronutrients significantly increases the productivity of species of the genus *Pleurotus*, particularly in the case of woody materials with poor mineral composition [17,31].

Phosphorus (P) occurred at the highest level in the commercial substrate (1.151 g·kg^−1^ DM), while among the tested raw materials the highest values were found in apple pomace (0.771 g·kg^−1^ DM) and spent coffee grounds (0.711 g·kg^−1^ DM). The lowest value was recorded in oak sawdust (0.159 g·kg^−1^ DM). This indicates that selected food-processing residues may contribute phosphorus to substrate mixtures, although their technological usefulness depends on the complete mixture composition and pH correction. The literature indicates that insufficient phosphorus content in the substrate may limit mycelial growth and the biological efficiency of *Pleurotus ostreatus*, whereas mineral supplementation increases yield [25,29].

Potassium (K) reached the highest concentration in wheat straw (13.776 g·kg^−1^ DM), a value higher than that recorded for the commercial substrate (12.195 g·kg^−1^ DM). Rapeseed straw was also characterized by high K content (8.014 g·kg^−1^ DM). Spent coffee grounds contained considerably less potassium (2.322 g·kg^−1^ DM); however, when incorporated into mixtures together with lignocellulosic materials, the final values may be higher. Narváez et al. documented changes in macronutrients, including potassium, during the growth of *Pleurotus ostreatus* in a compost based on sugarcane bagasse [32]. In the present study, potassium content is considered part of the mineral profile of the raw materials and proposed mixtures; its bioavailability and direct effect on mycelial growth or yield were not evaluated.

Calcium (Ca) occurred at the highest concentration in rapeseed straw (11.601 g·kg^−1^ DM), more than twice the value found in the commercial substrate (4.785 g·kg^−1^ DM). The high Ca content of this material may promote structural stability and buffering of the mixture pH. Apple pomace contained only small amounts of calcium (0.858 g·kg^−1^ DM), which, combined with its low pH, may increase the risk of substrate acidification.

Magnesium (Mg) reached the highest values in spent coffee grounds (1.772 g·kg^−1^ DM) and hemp shives (1.063 g·kg^−1^ DM), while the commercial substrate contained 1.570 g·kg^−1^ DM of Mg. This indicates the high potential of spent coffee grounds as a component supporting mycelial enzymatic activity. Bellettini et al. (2019) [17] emphasized that magnesium plays an important role as a cofactor of enzymes involved in lignocellulose degradation; therefore, its appropriate level may influence the intensity of substrate colonization. However, excessive Mg may reduce yield. Other authors [33] demonstrated that supplementation at the level of 2.1 g·kg^−1^ substrate significantly reduced biological efficiency compared with lower doses, indicating the need to maintain a moderate concentration of this element in the substrate.

Sodium (Na) occurred at low concentrations in all materials (0.000–0.195 g·kg^−1^ DM), which should be considered favorable from the perspective of minimizing the risk of substrate salinity. According to Sánchez, sodium concentrations above 1 g·kg^−1^ DM may negatively affect mycelial growth and yield, particularly under conditions of limited moisture [29]. The values obtained in the present study were several times lower than levels considered potentially unfavorable, and therefore did not indicate a salinity-related limitation under the adopted reference assumptions.

Compared with the commercial substrate, most raw materials exhibited lower concentrations of phosphorus and magnesium, whereas some straw materials exceeded it in potassium and calcium content. This indicates the possibility of balancing macronutrients through the appropriate selection of component proportions in the substrate by synergistically combining lignocellulosic materials with residues from agri-food processing.

The radar chart (Figure 3) illustrates the complementary mineral profiles of the tested materials. Wheat straw was distinguished by high K content, rapeseed straw by high Ca content, and spent coffee grounds by elevated Mg and P levels, indicating that component selection may support mineral balancing in candidate mixtures.

The content of microelements (Table 3, Figure 4) was differentiated. The literature indicates that both deficiency and excess of microelements may affect enzymatic activity and the biological efficiency of cultivated mushrooms [12]. These studies emphasize that a controlled level of microelements in the substrate is important for optimizing metabolism and the accumulation of elements in fruiting bodies.

Iron (Fe) reached the highest concentration in the commercial substrate (331.584 mg·kg^−1^ DM). Among the tested raw materials, the highest Fe content was found in oak sawdust (205.070 mg·kg^−1^ DM), whereas the lowest occurred in apple pomace (26.927 mg·kg^−1^ DM). Iron is an essential microelement and a component of oxidoreductase enzymes (including peroxidases) involved in lignin degradation [8].

Zinc (Zn) occurred at the highest levels in the commercial substrate (89.171 mg·kg^−1^ DM) and in spent coffee grounds (53.261 mg·kg^−1^ DM). Lignocellulosic materials contained Zn within the range of 8.837–44.527 mg·kg^−1^ DM. The Zn content in substrates for species of the genera *Pleurotus* and *Agaricus* usually falls within the range of 20–100 mg·kg^−1^ DM, and moderate supplementation may enhance the activity of oxidoreductase enzymes [12,34]. Therefore, the obtained values fall within the range typical of production substrates and do not indicate a risk of excessive accumulation of this element.

Copper (Cu) reached the highest concentration in spent coffee grounds (26.275 mg·kg^−1^ DM), whereas in the commercial substrate it amounted to 11.533 mg·kg^−1^ DM. In the remaining materials, its content ranged from 7.613 to 21.033 mg·kg^−1^ DM.

The concentrations of microelements in the analyzed raw materials were generally lower than those found in the commercial substrate, which may indicate the use of mineral supplementation in industrial substrate production.

The radar chart (Figure 4) confirmed differences in microelement profiles among the tested materials. The commercial reference substrate showed the highest Fe and Zn contents, while spent coffee grounds were characterized by the highest Cu content. These differences suggest that component selection may influence microelement availability, although biological verification is required to assess their actual effect on mycelial growth and yield.

The former Regulation of the Minister of Agriculture and Rural Development of 18 June 2008, which remained in force until 5 September 2024, specified permissible limits for heavy metal content in fertilizers and soil-improving products [35]. Although mushroom cultivation substrates are not classified as fertilizers, the values were adopted solely as a historical reference benchmark for assessing the chemical safety of the investigated materials (Table 4). The contents of the analyzed heavy metals (Cr, Ni, Cd, Hg, Pb, and As) in all samples did not exceed the historical reference limits adopted from that regulation. The determined concentrations remained within safe ranges, and in some samples the content of certain elements (including As) was below the method’s limit of detection (LOD). This is of considerable importance both from the perspective of fruiting body production safety and the potential utilization of spent substrate as an organic fertilizer.

The obtained results showed clear differences among the investigated materials in terms of nutrient proportions, which may indicate their potential functions in designing substrate mixtures for *Pleurotus ostreatus* cultivation. Lignocellulosic materials were characterized by high C:N ratios (80–257), significantly higher than that of the commercial substrate (49.05), suggesting their potential role as structural components; however, nitrogen supplementation would be required to obtain more favorable physicochemical conditions for further cultivation trials.

Among the base materials, triticale straw showed the C:N ratio closest to the functional target range (80.27). Wheat straw and rapeseed straw, due to their high potassium content (13.776 and 8.014 g·kg^−1^ DM, respectively), and in the case of rapeseed straw also a very high calcium content (11.601 g·kg^−1^ DM), may contribute to mineral balance and pH buffering in candidate mixtures. In practical terms, straw materials therefore constitute the most balanced group of base components.

For materials with the highest C:N ratios, such as sawdust and hemp shives, the incorporation of nitrogen-rich supplements (e.g., cereal bran or plant meal) could represent an effective strategy for improving nitrogen balance in designed substrate mixtures.

Spent coffee grounds, owing to the highest nitrogen content (21.610 g·kg^−1^ DM) and the lowest C:N ratio (25.96), appear to be a promising component for regulating the carbon-to-nitrogen ratio in candidate mixtures. Their high magnesium content further increases the nutritional value of the substrate. However, their use requires caution because excessive proportions may reduce mixture pH, increase the concentration of readily mineralizable compounds, and introduce phenolics, caffeine, and lipids that can influence microbial stability and contamination risk. For this reason, their use should be limited to controlled supplementation rather than as a dominant component.

Apple pomace was distinguished by a high phosphorus content (0.771 g·kg^−1^ DM); however, its low pH (3.85) limits the possibility of using it in larger proportions without prior pH adjustment.

The analysis of macro- and microelements indicates that waste-derived raw materials may constitute a potential source of mineral nutrients; however, compared with the commercial substrate, their levels were generally lower, which justifies the need for proper balancing of mixture composition. Appropriate component selection may support the development of mixtures with more favorable physicochemical conditions, including a C:N ratio within the adopted screening range, pH close to 5.0–6.5, and balanced macro- and microelement contents.

The content of heavy metals did not indicate exceedance of the adopted reference limits, supporting a preliminary chemical safety assessment of the investigated materials.

The graphical presentation of the results (Figure 2, Figure 3 and Figure 4), including both the investigated raw materials and the commercial substrate as a practical reference material, showed that the commercial reference substrate differed from the remaining materials due to its more balanced composition. It simultaneously exhibited a moderately high carbon content and an elevated nitrogen level, resulting in a favorable C:N ratio that is difficult to achieve when using only a single type of raw material. This indicates that its composition includes both lignocellulosic components performing a structural role and nitrogen-rich supplements increasing the nutritional value of the substrate. Therefore, candidate mushroom-growing media should consist of properly selected component combinations ensuring suitable physical structure and more favorable physicochemical conditions, which must still be verified in cultivation trials.

Taking into account the literature data and the performed dry-mass calculations, practical supplement proportions were selected for the preparation of candidate mixtures (Table 5). The Solver analysis with a maximum additive proportion of 20% identified wheat and triticale straw as base materials capable of reaching the adopted C:N screening range without exceeding the practical additive limit. Under the assumed screening criteria (C:N = 25–60, pH = 5.0–6.5, and supplement share ≤ 20% *w*/*w*), only these two base materials enabled the formulation of compositions meeting all criteria. Mixtures containing 25% and 30% spent coffee grounds were included solely as additional comparative variants to illustrate the additive proportions required for materials with higher initial C:N ratios. These variants exceeded the 20% limit and were not considered formulations meeting all screening criteria. The mixtures were prepared and their pH was experimentally determined according to the procedure described above. As reported by Chai et al. [27], increasing the proportion of spent coffee grounds as a supplement gradually slowed mycelial growth, whereas proportions exceeding 30% (*w*/*w*) inhibited fruiting. Supplementation up to 20% (*w*/*w*) did not significantly affect mycelial growth.

Table 5 presents only the results obtained for supplementation with spent coffee grounds. In the case of apple pomace, achieving the desired C:N ratio would require its application at a very high proportion in the mixture and would simultaneously lower pH below the adopted target range, which may make this option unsuitable without pH correction.

Many studies have applied mixture design approaches to develop substrates for mushroom cultivation in order to determine the optimal proportions of components [36,37,38,39]. In the present study, the Solver-based approach should be interpreted as a preliminary screening step that supports the selection of candidate mixtures, rather than as confirmation of technological suitability. The adopted C:N range was therefore used only as a physicochemical screening criterion and should not be interpreted as evidence of optimal cultivation performance.

Among the analyzed variants, the most promising physicochemical profiles were obtained for mixtures based on straw materials, particularly triticale straw and wheat straw supplemented with spent coffee grounds. These materials were characterized by relatively stable pH and a moderate requirement for supplementation, which allowed mixtures to be formulated while maintaining expected structural properties. The obtained results provide a basis for further experimental studies, especially cultivation trials assessing colonization rate, yield, and biological efficiency.

The obtained results indicate that none of the analyzed raw materials individually fulfilled all adopted physicochemical criteria for *Pleurotus ostreatus* substrate formulation. However, their complementary properties provide a basis for developing candidate mixtures, especially those based on wheat or triticale straw supplemented with spent coffee grounds. These mixtures require further validation in terms of mycelial colonization, yield, biological efficiency, and contamination risk.

A limitation of the present study is the lack of lignocellulosic-fraction analysis, including lignin, cellulose, and hemicellulose contents. These analyses were not included because the present stage focused on preliminary elemental and physicochemical characterization and the screening of possible mixtures; their determination is planned for the next research stage. Physical substrate properties such as porosity, water-holding capacity, particle-size distribution, and structural stability were also not assessed. Therefore, the results support only the preliminary selection of candidate components and mixtures. A comprehensive assessment requires lignocellulosic-fraction analysis, physical characterization, and cultivation trials evaluating mycelial colonization, yield, and biological efficiency.

## 4. Conclusions

Lignocellulosic materials such as sawdust, straws, and hemp shives were characterized by high C:N ratios (80–257) and low nitrogen content (2.04–5.85 g·kg^−1^ DM), indicating their potential structural function and the need for nitrogen supplementation in candidate mixtures.

The use of nitrogen-rich supplements could support the utilization of materials with high C:N ratios by improving the nitrogen balance of formulated candidate substrates.

Spent coffee grounds, owing to the highest nitrogen content (21.61 g·kg^−1^ DM) and the lowest C:N ratio (25.96), were the most promising tested supplement for regulating the carbon and nitrogen balance. However, their excessive proportion may reduce mixture pH and increase contamination risk; therefore, controlled dosing and cultivation validation are required.

Apple pomace contributed phosphorus (771 mg·kg^−1^ DM), but its low pH (3.85) limits its direct application without prior pH correction.

Wheat straw and rapeseed straw, due to their high potassium (up to 13.8 g·kg^−1^ DM) and calcium contents (up to 11.6 g·kg^−1^ DM), may contribute to mineral balance and pH buffering in candidate mixtures.

The content of microelements and heavy metals did not indicate exceedance of the adopted reference limits, supporting a preliminary chemical safety assessment of the investigated materials.

The results indicate that none of the tested plant-derived residues alone fulfilled all assumed criteria. However, wheat and triticale straw supplemented with spent coffee grounds showed the most promising physicochemical profiles for preliminary selection. Lignocellulosic-fraction analysis, physical characterization, and cultivation trials are required to verify substrate performance, mycelial colonization, yield, and biological efficiency.

## Figures and Tables

**Figure 1:**
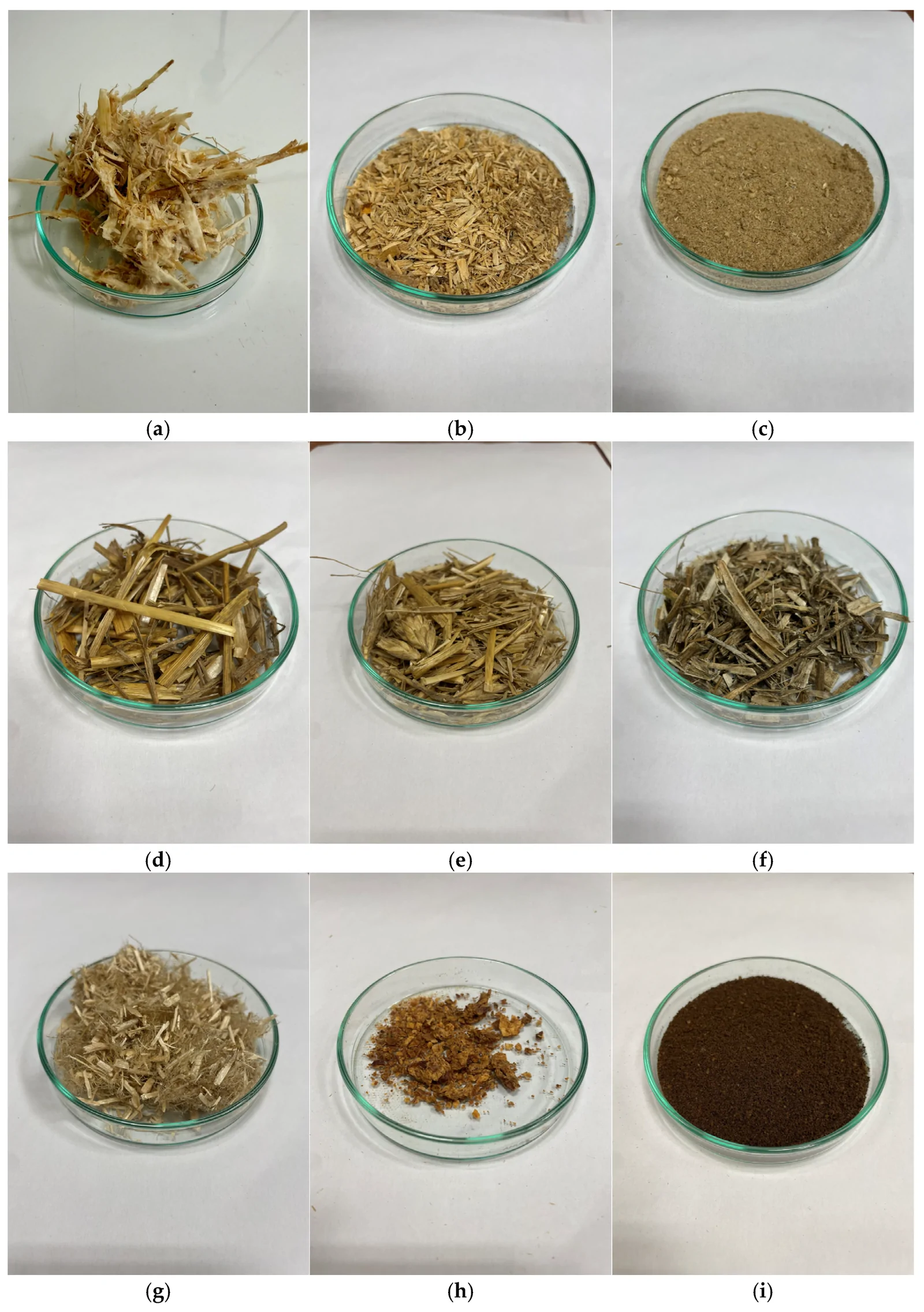
Materials investigated in the study (own photographs).

**Figure 2:**
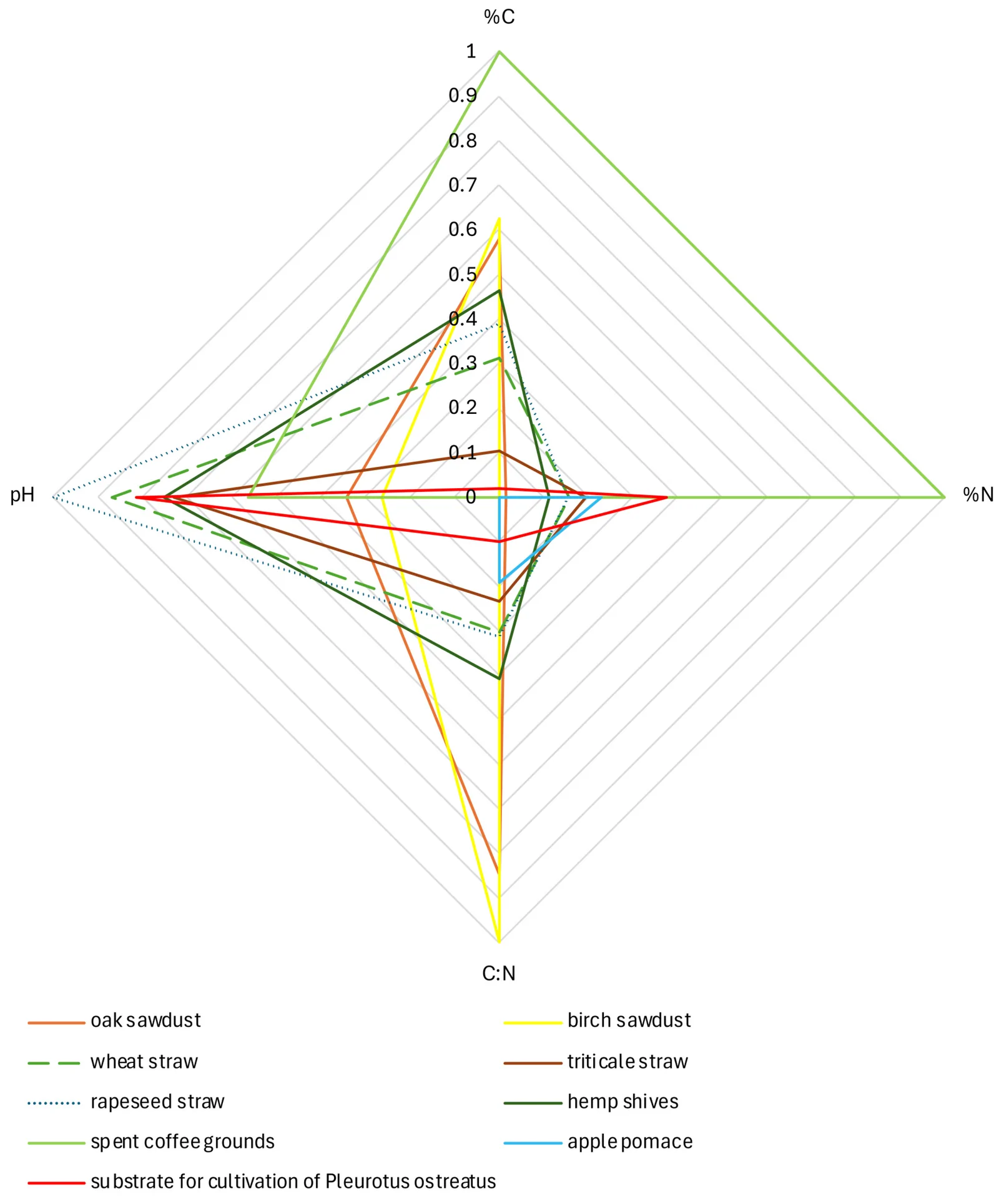
Radar chart showing the min–max-normalized values of C, N, C:N ratio, and pH of the investigated materials. Each variable was transformed to a 0–1 scale, where 0 and 1 represent the lowest and highest values observed in the analyzed dataset, respectively. The normalized values describe relative physicochemical profiles and should not be interpreted as substrate-suitability scores.

**Figure 3:**
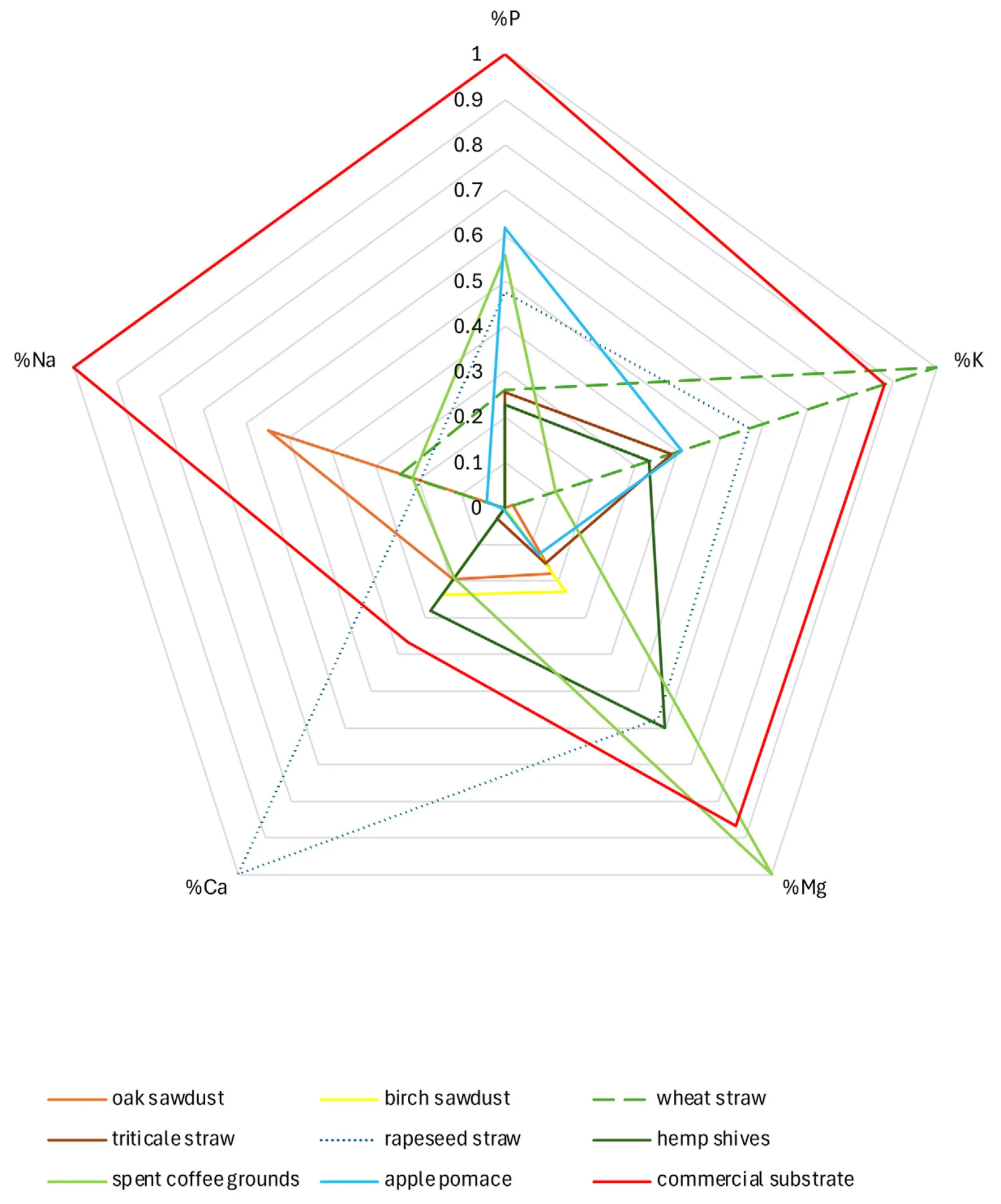
Radar chart of normalized macroelement contents (P, K, Mg, Ca, and Na) in the analyzed materials.

**Figure 4:**
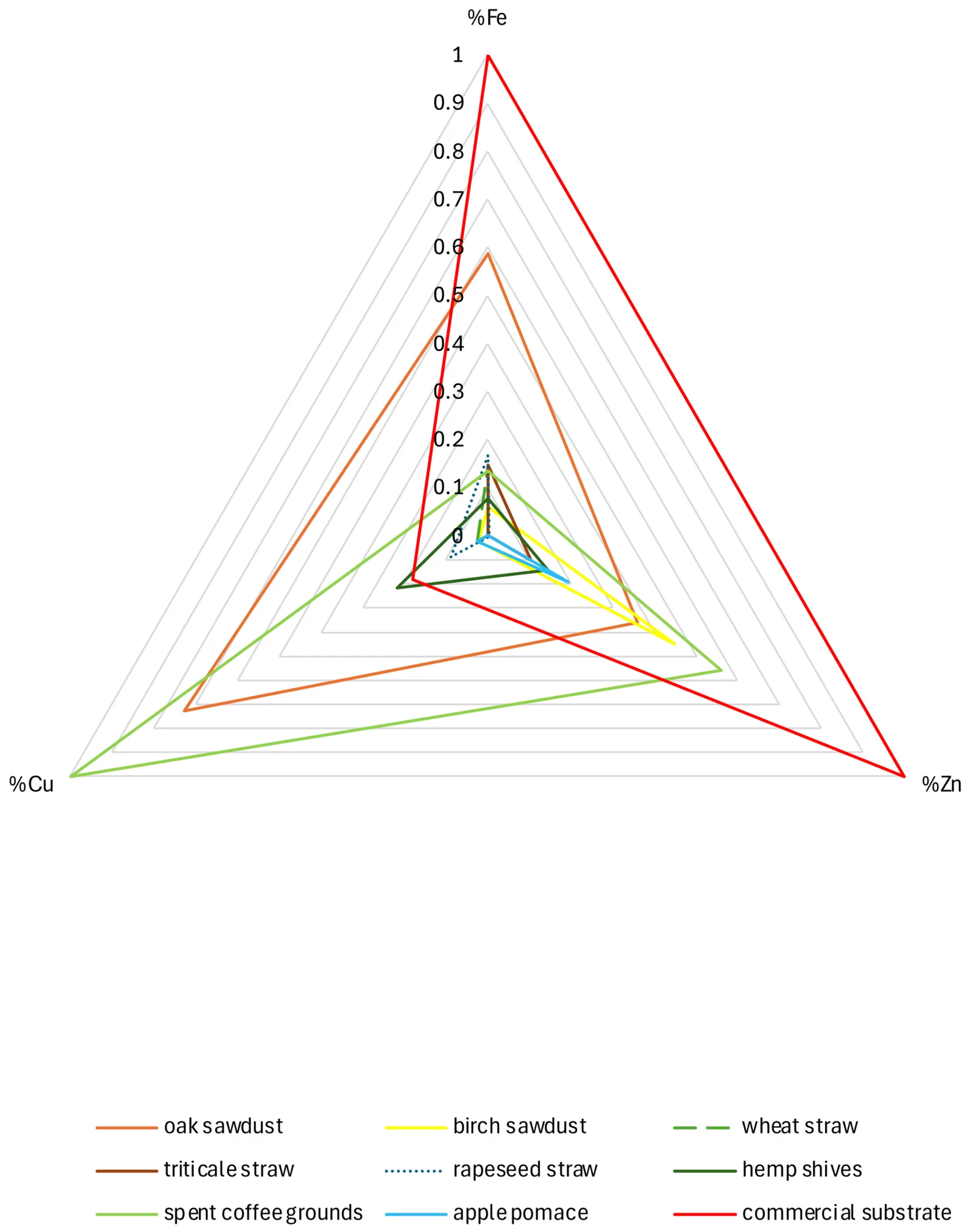
Radar chart of normalized microelement contents (Fe, Zn, and Cu) in the analyzed materials.

**Table 1:** Content of C, N, C:N ratio, and pH of the investigated materials.

Material	Type	C [g·kg^−1^ DM]	N [g·kg^−1^ DM]	C:N	pH
**oak sawdust**	**base** **component**	518.200 ± 1.598 ^ab^	2.340 ± 0.004 ^f^	221.453	4.63 ± 0.007 ^cd^
**birch sawdust**		522.800 ± 0.283 ^ab^	2.035 ± 0.025 ^f^	256.904	4.45 ± 0.007 ^de^
**wheat straw**		490.950 ± 0.460 ^bcd^	5.269 ± 0.052 ^de^	93.177	5.84 ± 0.170 ^a^
**triticale straw**		469.550 ± 2.623 ^cd^	5.850 ± 0.020 ^cd^	80.265	5.52 ± 0.311 ^ab^
**rapeseed straw**		498.600 ± 0.891 ^bcd^	5.080 ± 0.030 ^de^	98.150	6.14 ± 0.233 ^a^
**hemp shives**		506.300 ± 0.552 ^bc^	4.220 ± 0.006 ^e^	119.976	5.57 ± 0.247 ^ab^
**spent coffee grounds**	**supplement**	561.000 ± 0.184 ^a^	21.610 ± 0.008 ^a^	25.960	5.14 ± 0.014 ^bc^
**apple pomace**		459.100 ± 0.311 ^d^	6.525 ± 0.001 ^c^	70.360	3.85 ± 0.000 ^e^
**substrate for cultivation of *Pleurotus*** ***ostreatus***	**commercial** **substrate**	461.050 ± 0.120 ^d^	9.400 ± 0.027 ^b^	49.048	5.71 ± 0.042 ^ab^

Within each column, different superscript letters indicate statistically different values according to post hoc comparison (Tukey’s test) at *p* ≤ 0.05. Values are expressed as mean ± SD (n = 3 analytical replicates). The C:N ratio was calculated from the mean C and N contents, whose standard deviations are reported separately; the derived ratio was used descriptively, and no separate standard deviation or statistical analysis was applied.

**Table 2:** Content of macronutrients (P, K, Mg, Ca, Na) in the analyzed materials.

Material	Type	P	K		Mg	Ca	Na
		[g·kg^−1^ DM]					
**oak sawdust**	**base component**	0.159 ± 0.011 ^d^	1.019 ± 0.004 ^d^	0.483 ± 0.003 ^c^		2.904 ± 0.000 ^c^	0.195 ± 0.002 ^b^
**birch sawdust**		0.302 ± 0.006 ^cd^	0.782 ± 0.005 ^d^	0.557 ± 0.004 ^c^		3.384 ± 0.062 ^bc^	ND
**wheat straw**		0.419 ± 0.096 ^c^	13.776 ± 0.002 ^a^	0.204 ± 0.006 ^c^		0.822 ± 0.014 ^d^	0.086 ± 0.001 ^bc^
**triticale straw**		0.413 ± 0.025 ^c^	5.618 ± 0.094 ^c^	0.446 ± 0.009 ^c^		1.145 ± 0.030 ^d^	0.000 ± 0.000 ^c^
**rapeseed straw**		0.631 ± 0.066 ^b^	8.014 ± 0.044 ^b^	1.024 ± 0.019 ^b^		11.601 ± 0.098^a^	0.070 ± 0.001 ^c^
**hemp shives**		0.386 ± 0.076 ^c^	4.936 ± 0.022 ^c^	1.063 ± 0.010 ^b^		3.849 ± 0.008 ^bc^	0.000 ± 0.001 ^c^
**spent coffee grounds**	**supplement**	0.711 ± 0.043 ^b^	2.322 ± 0.008 ^d^	1.772 ± 0.006 ^a^		2.860 ± 0.006 ^c^	0.076 ± 0.004 ^c^
**apple pomace**		0.771 ± 0.010 ^b^	5.954 ± 0.026 ^c^	0.399 ± 0.008 ^c^		0.858 ± 0.019 ^d^	0.015 ± 0.001 ^c^
**substrate for cultivation of *Pleurotus*** ***ostreatus***	**commercial** **substrate**	1.151 ± 0.025 ^a^	12.195 ± 0.052 ^a^	1.570 ± 0.017 ^a^		4.785 ± 0.001 ^b^	0.355 ± 0.008 ^a^

Within each column, different superscript letters indicate statistically different values according to post hoc comparison (Tukey’s test) at *p* ≤ 0.05. Values are expressed as mean ± SD. ND—not detected under the conditions of the analytical method used.

**Table 3:** Content of microelements (Fe, Zn, Cu) in the investigated materials.

Material	Type	Fe	Zn	Cu
		[mg kg^−1^ DM]		
**oak sawdust**	**base component**	205.070 ± 0.242 ^b^	37.369 ± 0.070 ^b^	21.033 ± 0.075 ^ab^
**birch sawdust**		45.224 ± 0.035 ^cd^	44.527 ± 0.034 ^ab^	7.999 ± 0.032 ^b^
**wheat straw**		71.417 ± 0.001 ^cd^	8.837 ± 0.010 ^b^	8.084 ± 0.002 ^b^
**triticale straw**		72.187 ± 0.074 ^cd^	16.141 ± 0.034 ^b^	7.613 ± 0.014 ^b^
**rapeseed straw**		77.920 ± 0.063 ^c^	9.047 ± 0.010 ^b^	9.260 ± 0.022 ^b^
**hemp shives**		50.891 ± 0.098 ^cd^	20.234 ± 0.139 ^b^	12.216 ± 0.074 ^ab^
**spent coffee grounds**	**supplement**	67.965 ± 0.164 ^cd^	53.261 ± 0.146 ^ab^	26.275 ± 0.043 ^a^
**apple pomace**		26.927 ± 0.025 ^d^	24.059 ± 0.052 ^b^	8.093 ± 0.006 ^b^
**substrate for cultivation of *Pleurotus ostreatus***	**commercial** **substrate**	331.584 ± 0.139 ^a^	89.171 ± 0.267 ^a^	11.533 ± 0.042 ^ab^

Within each column, different superscript letters indicate statistically different values according to post hoc comparison (Tukey’s test) at *p* ≤ 0.05. Values are expressed as mean ± SD.

**Table 4:** Content of heavy metals (Cr, Ni, Cd, Hg, Pb, As) in the analyzed materials.

Material	Type	Cr	Ni	Cd	Hg	Pb	As
		[mg·kg^−1^ DM]					
**oak sawdust**	**base** **component**	11.144 ± 0.771	0.916 ± 0.607	0.150 ± 0.001	0.010 ± 0.001	3.789 ± 0.003	0.060 ± 0.002
**birch sawdust**		0.528 ± 0.019	ND	0.231 ± 0.017	0.005 ± 0.001	1.438 ± 0.025	ND
**wheat straw**		0.491 ± 0.160	ND	0.357 ± 0.004	0.008 ± 0.007	1.064 ± 0.382	0.011 ± 0.011
**triticale straw**		0.496 ± 0.146	ND	0.284 ± 0.234	0.001 ± 0.000	0.721 ± 0.073	0.238 ± 0.336
**rapeseed straw**		3.445 ± 0.349	2.864 ± 0.004	0.250 ± 0.055	ND	0.805 ± 0.016	ND
**hemp shives**		0.831 ± 0.120	1.831 ± 0.085	2.550 ± 0.048	0.007 ± 0.001	0.597 ± 0.046	ND
**spent coffee grounds**	**supplement**	0.235 ± 0.023	3.107 ± 0.267	3.938 ± 0.043	0.089 ± 0.094	0.982 ± 0.196	ND
**apple pomace**		0.332 ± 0.056	ND	0.017 ± 0.006	0.013 ± 0.001	0.473 ± 0.130	ND
**substrate for cultivation of *Pleurotus ostreatus***	**commercial** **substrate**	0.037 ± 0.052	0.652 ± 0.198	0.169 ± 0.005	0.003 ± 0.000	1.563 ± 0.506	0.711 ± 0.116

Note: ND = not detected under the conditions of the analytical method used.

**Table 5:** Calculated C:N ratios and experimentally determined pH values of selected substrate mixtures supplemented with spent coffee grounds. Variants containing ≤20% spent coffee grounds met the adopted additive criterion; variants containing 25% and 30% were included only for comparison and did not meet all screening criteria.

Material	Supplement Amount (Spent Coffee Grounds)	C:N	pH
	[% *w*/*w*]		
**triticale straw**	20	54.19	5.94
**wheat straw**	20	59.15	6.03
**rapeseed straw**	25	55.82	6.27
**hemp shives**	30	55.39	5.35

## Data Availability

The original contributions presented in this study are included in the article. Further inquiries can be directed to the corresponding author.

## Abbreviations

The following abbreviations are used in this manuscript:

AAS	atomic absorption spectrophotometry
C:N	carbon to nitrogen ratio
CE	circular economy
DM	dry matter
ICP-MS	inductively coupled plasma mass spectrometry
LOD	limit of detection
TOC	total organic carbon
